# Experimentally testing mate preference in an avian system with unidirectional bill color introgression

**DOI:** 10.1002/ece3.9812

**Published:** 2023-02-21

**Authors:** Callum S. McDiarmid, Fiona Finch, Marianne Peso, Erica van Rooij, Daniel M. Hooper, Melissah Rowe, Simon C. Griffith

**Affiliations:** ^1^ School of Natural Sciences Macquarie University Sydney New South Wales Australia; ^2^ Department of Biological Sciences Columbia University New York New York USA; ^3^ Institute for Comparative Genomics American Museum of Natural History New York New York USA; ^4^ Department of Animal Ecology Netherlands Institute of Ecology (NIOO‐KNAW) Wageningen The Netherlands

**Keywords:** avian, bill color, hybrid zone, introgression, mate preference

## Abstract

Mating behavior can play a key role in speciation by inhibiting or facilitating gene flow between closely related taxa. Hybrid zones facilitate a direct examination of mating behavior and the traits involved in establishing species barriers. The long‐tailed finch (*Poephila acuticauda*) has two hybridizing subspecies that differ in bill color (red and yellow), and the yellow bill phenotype appears to have introgressed ~350 km eastward following secondary contact. To examine the role of mate choice on bill color introgression, we performed behavioral assays using natural and manipulated bill colors. We found an assortative female mating preference for males of their own subspecies when bill color was not manipulated. However, we did not find this assortative preference in trials based on artificially manipulated bill color. This could suggest that assortative preference is not fixed entirely on bill color and instead may be based on a different trait (e.g., song) or a combination of traits, or alternatively may be due to lower statistical power alongside the bill manipulations being unconvincing to the female choosers. Intriguingly, we find a bias in the inheritance of bill color in captive bred F1 hybrid females. Previous modeling suggests that assortative mate preference and this kind of partial dominance in the underlying genes may together contribute to introgression, making the genetic architecture of bill color in this system a priority for future research.

## Graphical abstract

We investigate whether mate choice might play a role in the unidirectional introgression of yellow bill color between subspecies of the long‐tailed finch. Females demonstrate an assortative mate preference for unmanipulated males of their own subspecies. However, the evidence that females base this assortative preference on bill color is equivocal.

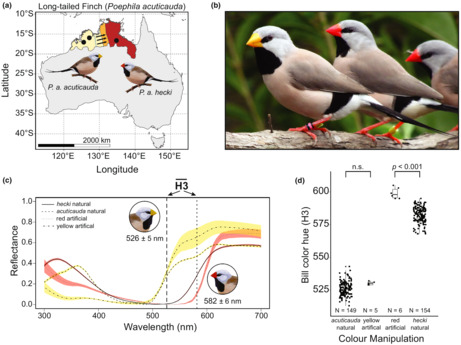

## 1 INTRODUCTION

For many animals, recognizing and attracting mates of their own species is a critically important task (Ryan & Rand, 1993; Servedio & Boughman, 2017). Hybrid zones are powerful natural systems with which to examine the role of mate choice, and the traits involved in evaluating mate quality, in the emergence of reproductive isolation (Coyne & Orr, 2004; Hudson & Price, 2014; Irwin, 2020; Price, 2008). While it is commonly thought that sexual selection acting within a lineage, that is, the evolution of novel sexual traits and preferences for them, can lead to assortative mating between lineages, such outcomes are not guaranteed (Ritchie, 2007). For example, if the forces of selection by which a novel trait spreads to fixation within one population are shared among populations then sexual selection will act against population divergence (Miñano et al., 2021). Indeed, the traits that distinguish hybridizing lineages appear to move across hybrid zones with an appreciable frequency (Baldassarre et al., 2014; Lipshutz et al., 2019; Parsons et al., 1993; While et al., 2015). Experimental evaluation of the strength and targets of sexual selection is, therefore, a key component for understanding the evolution of reproductive isolation and patterns of introgression in hybrid systems.

Hybridization can facilitate the sharing of beneficial genetic mutations and phenotypes that accumulated in parental lineages during periods of isolation (Abbott et al., 2013; Harrison, 1986; Marques et al., 2019; Wu, 2001). The extent of introgression will be influenced by the fitness implications of these novel gene combinations and resulting phenotypes, together with the local recombination rate, epistatic interactions, and demography (Barton & Bengtsson, 1986; Schumer et al., 2018; Wu, 2001). Traits that reduce fitness in the receiving lineage will show resistance to introgression, and act to limit the introgression of linked genetic material, whereas unlinked selectively neutral alleles may flow unopposed between hybridizing lineages (Barton, 1979; Gompert et al., 2012; Harrison, 1990; Payseur, 2010). In contrast, theory predicts that strong positive selection can drive the introgression of adaptive genotypes and phenotypes between lineages (Baldassarre et al., 2014; Barton, 2001; Hedrick, 2013; Morjan & Rieseberg, 2004). Indeed, most cases of unidirectional introgression of color traits that have been studied in detail have concluded that they were likely adaptive, resulting from fitness benefits due to aposematism or sexual selection (Baldassarre et al., 2014; MacGregor et al., 2016; Pardo‐Diaz et al., 2012; Stein & Uy, 2006; While et al., 2015).

Mate choice could contribute to unidirectional introgression of phenotypic traits through two main mechanisms. Firstly, if a novel trait emerges in one population but a preference for it exists across all populations, we would expect selection to result in the introgression of this trait between populations following secondary contact, so long as the allelic variation for the novel trait is not incompatible with standing variation in recipient populations (Wirtz, 1999). This phenomenon appears to be driving introgression of the golden collars from golden collared manakins into the white‐collared species (Stein & Uy, 2006), and the introgression of red back plumage into orange‐backed subspecies in the red‐backed fairy wren (Baldassarre et al., 2014; Baldassarre & Webster, 2013). Secondly, and somewhat counterintuitively, two recent studies of avian hybrid zones employed simulations to reveal that assortative mating can also result in unidirectional introgression, when combined with epistasis or dominance among underlying genes (Metzler et al., 2021; Semenov et al., 2021). These cases demonstrate that mate choice can be important for introgression, but this remains relatively understudied, so additional data on species in contact will provide a broader understanding of the evolutionary forces underlying unidirectional introgression.

**FIGURE 1:**
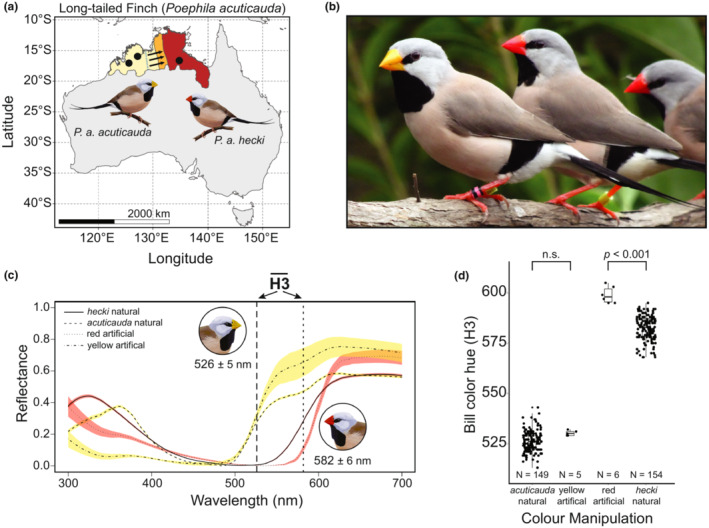
Bill color differentiation between subspecies of the long‐tailed finch (*Poephila acuticauda*). (a) Approximate geographic distribution of bill color variation across the range of the long‐tailed finch from yellow bills in the west, red bills in the east, and a narrow region of orange bills in between following from Griffith and Hooper (2017). The location of primary genomic admixture (Z chromosome and mtDNA) between subspecies is represented as a dashed blue line following from Hooper et al. (2019) and Lopez et al. (2021) with arrows representing the hypothesized direction of introgression of yellow bill color from west to east. The location of source populations of birds used in this study are represented on the map as black circles (see Methods for precise locations). (b) Representative photograph of long‐tailed finches from our research colony used in this study: from left, subspecies *acuticauda*, subspecies *hecki*. (c) Standardized reflectance spectra for bills from individuals of subspecies *acuticauda* (*N* = 71) and *hecki* (*N* = 80), as well as red (*N* = 5) and yellow (*N* = 5) artificial bill manipulations, are shown as mean and standard error. The mean and standard deviation for bill hue (colorimetric variable H3; the wavelength midway between maximum and minimum reflectance between 400 and 700 nm) is shown for each subspecies as vertical lines. (d) Natural variation in bill color hue (H3) for individuals of each subspecies compared against our artificially manipulated yellow and red treatments. Our artificial yellow manipulation overlaps the range of variation observed in natural *acuticauda* bills but our artificial red manipulation is redder than the range of variation observed in natural *hecki* bills. Different letters indicate statistical significance between treatment groups (*p* < 0.05; Tukey's HSD test).

The long‐tailed finch *Poephila acuticauda* is an Australian estrildid finch characterized by a yellow‐billed subspecies in the West (*P. a. acuticauda*) and a red‐billed subspecies in the East (*P. a. hecki*) that come into secondary contact on the eastern edge of the Kimberley Plateau (Figure 1a, Hooper et al., 2019). In addition to their difference in bill color, subspecies are also distinct in their nuclear and mitochondrial genomes (Hooper et al., 2019; Lopez et al., 2021), and to some degree in their songs (Zann, 1976) and sperm size (Rowe et al., 2015). The respective geographic centers of genetic and bill color admixture are significantly displaced from each other: the hybrid zone for bill color (as well as three candidate genes underlying variation in this trait) is located 350 km east of the hybrid zone for the vast majority of the genome (Hooper et al., 2019; Lopez et al., 2021). Genetic analyses suggest that variation for yellower bills has introgressed from subspecies *acuticauda* into subspecies *hecki* (Hooper et al., 2019; Lopez et al., 2021). Given that bill color in the long‐tailed finch is an oligogenic trait—that is, heritable variation is seemingly governed by only a small number of genes—(Hooper et al., 2019) and has a steep phenotypic cline, it is unlikely that stochasticity alone is responsible for this apparent unidirectional introgression of bill color. Instead, these findings suggest that introgression of the yellow billed phenotype may have an adaptive basis and so increase fitness in some way (Barton, 2001; Harrison & Larson, 2016). We hypothesize that precopulatory mate‐choice may increase fitness associated with yellow bill color and thus may be responsible for the unidirectional introgression observed in the long‐tailed finch, as observed in other birds (Baldassarre & Webster, 2013; Stein & Uy, 2006). There is little variation in bill color within allopatric populations (Griffith & Hooper, 2017), and a previous study that assessed assortative mating in one such allopatric population with yellow bills found no evidence of preference for the limited within‐population variation in bill coloration (van Rooij & Griffith, 2012). Here, we are not looking at sexual selection for an ornamental trait that varies significantly across the males within a single population (i.e. Gustafsson et al., 1995), but rather considering selection on a trait (red vs. yellow bills) that varies across a (sub‐) species boundary, that is, a species indicator trait (Sætre et al., 1997).

Here, we describe behavioral trials conducted on wild‐caught and first‐generation captive long‐tailed finches of both subspecies. Both natural and experimentally manipulated bill colors were used. Given that the genetic architecture of traits (e.g., dominance and epistasis) can play an important role in trait inheritance and the dynamics of introgression following hybridization, we also examined bill color of captive‐bred first generation (F_1_) hybrids to gain insight into the possible role of these processes in the long‐tailed finch system.

## 2 METHODS

### 2.1 Study animals

Long‐tailed finches used in these behavioral trials were either wild‐caught or the first‐generation pure‐bred offspring of wild‐caught individuals of each subspecies (*P. a. acuticauda* or *P. a. hecki*) held in aviaries at Macquarie University in Sydney, Australia. Wild‐caught *P. a. acuticauda* (Western subspecies, yellow billed) individuals were sourced from two populations in Western Australia: Mount House (17°02′S, 125°35′E) and Nelson's Hole (15°49′S, 127°30′E). Wild‐caught *P. a. hecki* (Eastern subspecies, red billed) individuals were sourced from October Creek, Northern Territory (16°37′S, 134°51′E; Figure 1). All birds used in mate choice trials were sexually mature (over 180 days post hatch) and were maintained during trials under breeding conditions; exposure to the opposite sex and ad libitum access to food and water. Trials were performed in 2011–2012 (by FF, MP and EvR), but were not written up for publication at that time. Given subsequent findings about the long‐tailed finch hybrid zone, particularly the apparent introgression of color (Hooper et al., 2019; Lopez et al., 2021), we revisit and analyze these data here to assess the role mate choice might have in bill color introgression.

### 2.2 Bill color manipulations

To experimentally determine whether any observed preference between subspecies is due to differences in bill color, for some trials the stimulus bird bill colors were manipulated using nail polish to be either yellow (Rimmel London O55 Sunshine), red (Rimmel London 310 Red), black (Rimmel London 800 Black out), or transparent (our control treatment revealing the underlying bill color of each subspecies). These red and yellow nail polish colors were selected as they appeared to be the closest match to the bill colors of each subspecies by the human eye. To assess how effective our bill manipulations were we used reflectance spectrophotometry to measure bill color of both unmanipulated individuals and individuals with manipulated bills, following published protocols (Griffith & Hooper, 2017; McDiarmid et al., 2022; van Rooij & Griffith, 2012). Spectral reflectance of the center of the upper mandible was recorded three times using a USB2000 + Miniature Fiber Optic spectrophotometer (Ocean Optics Inc., Dunedin, FL, USA), a xenon light source PX‐2 (Ocean Optics Inc.) with a fiber‐optic cable held at an angle of 90° to the bill and about 10 mm from the surface of the mandible with a plastic sheath that excluded ambient light. Reflectance data were captured using the AVASOFT 7 program (Avantes, Eerbeek, the Netherlands). To average and smooth the three reflectance spectra replicates for each individual we used Pavo 2 (Maia et al., 2019). The spectra were then normalized by maximum and minimum reflectance values, and we extracted the colorimetric variable H3 (a measure of bill hue extracted from spectrophotometer readings and represented as the wavelength midway between the minimum and maximum reflectance of a surface, (see Figure 1c; Maia et al., 2019)). This colorimetric variable effectively differentiates the bill colors of our two subspecies (H3: *acuticauda* mean = 526.3 ± 5.3 nm, *N* = 149; *hecki* mean = 582.4 ± 6.0 nm, *N* = 154; Figure 1d).

We compared the hue of manipulated bills to the range of variation observed in natural bills of each subspecies. The hue of the artificial ‘yellow’ manipulation was not significantly different from the natural yellow color of *acuticauda* bills (median H3: yellow = 530 nm [*N* = 5], *acuticauda* = 526 nm [*N* = 149]; permutation test: Z = −1.7, *p* = 0.090) and the effect size of this difference is small (4 nm difference in medians and 0.17 standardized effect size; Figure 1d). The hue of our artificial ‘red’ is significantly different and red‐shifted from the natural red color of *hecki* bills (median H3: red = 598 nm [*N* = 6], *hecki* = 583 nm [*N* = 154]; permutation test: Z = −5.9, *p* < 0.001) and the effect size of this difference is notable (15 nm difference in medians and 0.33 standardized effect size; Figure 1d). In other studies manipulating animal color signals in both the visual and ultra‐violet (UV) part of the spectrum has proved a challenge, with manipulations typically not closely matching a natural reflectance curve (e.g., Sheldon et al., 1999). Similarly here the natural bill color contains some variation in the UV part of the spectrum between subspecies that we were unable to capture with our manipulations (see Figure 1c). As such our manipulations are principally limited to the visual spectrum. As animals integrate visual signals from the visual (400–700 nm) and UV (300–400 nm) parts of the spectrum together in natural signals (Vorobyev & Osorio, 1998), experimental manipulations can still be effective for the purposes of testing the role of color signals as long as any noise in the UV signal does not overwhelm that in the visual part of the spectrum. To examine whether our metric H3 was capturing variation across both the UV and visual spectrums, we performed a principal component analysis (PCA) of the smoothed and normalized spectral data (as above) in both the UV and visual spectrum (300–700 nm) from natural and manipulated red and yellow birds (4 of each group, total 16 individuals). The first two principal components (PC1 and PC2) accounted for over 80% of total variance (Figure A4, Table A4), so we then examined whether variation in H3 was accounted for by PC1 and PC2 (by running a linear model with H3 as the response variable and PC1 and PC2 as fixed effects). PC1 and PC2 explained a large amount of the variation described in H3 (*R* ^2^ = 0.83). This suggests that our use of H3 is capturing the bulk of the main spectral variation and should therefore appropriately represent the yellow and red phenotypes. The ‘black’ manipulation was intended to mimic the coloration of juveniles, and the manipulation successfully resulted in a uniform and low reflectance across the avian color spectrum. There was no significant difference between the hues of bills prior to and following treatment with our clear control manipulation (paired t test: *t* = 1.5, *p* = 0.2385, *n* = 4).

### 2.3 Four‐way mate‐choice trials

The first set of trials performed exposed a focal (choosing) bird to four stimulus birds, all with manipulated bill colors. We describe the methodology and results of these ‘four‐way trials’ for completeness and transparency, however, they have issues (outlined below) that limit the interpretation of these trials. A second set of trials (‘two‐way trials’) with refined methodology were subsequently performed and we will focus on those.

**FIGURE A1:**
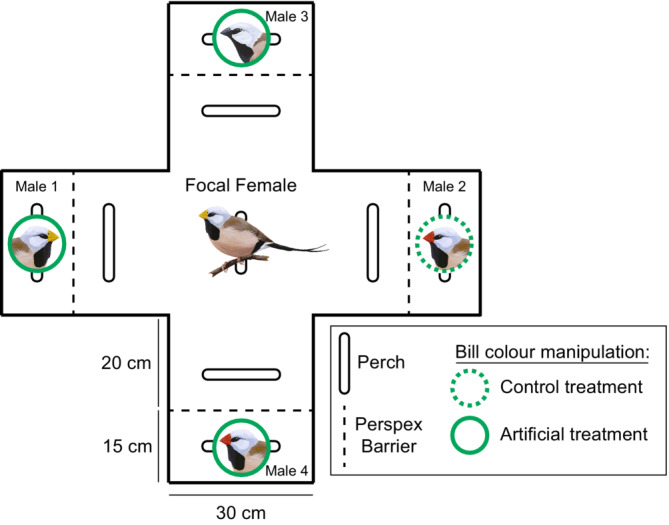
Design of mate association preference testing apparatus used in four‐way trials. In this scaled four‐way example, a female focal individual, center, has a choice between spending time perched in front of any of four male stimulus birds, each housed in their own unique arm. In all four‐way trials, three individuals were manipulated to have artificially yellow, red, or black colored bills. A fourth stimulus individual had clear nail polish applied to their bill, allowing their natural bill color to be shown (in this example, Male 2 has a natural *hecki* red bill). For the two‐way trials, two of the arms were blocked off (e.g., those with Male 3 and Male 4 here). Dashed lines represent Perspex barriers between each stimulus and the focal test individual. Note that bird illustrations are not to the scale of the mate association preference testing arena.

The four‐way mate choice trials were performed in a four‐armed wooden preference apparatus with the focal (choosing) bird starting on a perch in the middle (Figure A1). Each arm contained a perch in front of Plexiglass (Perspex; Plexiglas®, Röhm, Germany) following (Pryke & Griffith, 2007), behind which there was a stimulus bird of the opposite sex to the focal bird. These trials were performed with both male and female focal birds. All four stimulus birds had bill color manipulations (as described above), with each trial including one each of (1) yellow, (2) red, (3) black, or (4) clear. All trials were recorded with a video camera (Sony digital camcorder model DCR‐HC96), that filmed the central focal bird from above. The location of the focal bird was filmed for 60 min and scored for 45 min after the focal bird first visited a stimulus bird (sitting on the perch or floor adjacent to that arm of the apparatus). If the focal bird did not move for 15 min, the trial was not included in further analysis. Further details can be found in Supporting Information.

The limitations of these four‐way trials included (1) low sample sizes, due to the logistical difficulty of manipulating bill color (and removing those bill manipulations), exacerbated by the fact that trials had to be further subdivided by whether the ‘clear’ bill manipulation bird had a naturally red or yellow bill color; (2) the complicated design and low sample sizes meant that potentially confounding factors cannot be statistically accounted for (e.g., stimulus birds were included in variable numbers of trials, there was asymmetry in the genetic lineage of stimulus birds), and (3) the number of stimuli in the design meant that focal birds may have experienced sensory overload and were unable to express a clear preference. This is consistent with other laboratory groups studying avian behavior that transitioned away from four‐way trials around the time these were performed (e.g., the Forstmeier group: four‐way trials in Forstmeier and Birkhead 2004, Forstmeier 2004; both four and two‐way in: Schielzeth et al. 2008a, 2008b; two‐way trials in Schielzeth et al. 2010, Wang et al. 2017).

### 2.4 Two‐way female mate‐choice trials

**FIGURE 2:**
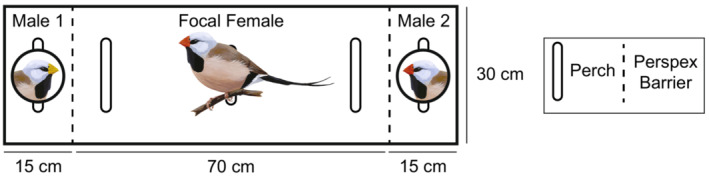
Design of mate association preference testing apparatus used in two‐way trials. In this scaled two‐way example, a focal female individual, center, has a choice between spending time perched in front of two male stimulus birds, each housed at opposite ends of the testing arena. Females were given a choice between two male stimuli from each subspecies with natural bill color or two male stimuli of the females' subspecies with manipulated bill color: that is, artificial yellow or red. Preference was evaluated as the proportion of time spent perched in front of either male. Dashed lines represent Perspex barriers between each stimulus and the focal test individual. Note that bird illustrations are not to the scale of the mate association preference testing arena.

The second set of trials (two‐way trials) were performed using only female focal birds, who were provided just two stimulus males at a time, with separate trials for manipulated and unmanipulated stimulus bill colors and an increased overall sample size. For these two‐way trials the existing four‐way testing apparatus was modified by blocking off two opposite arms (Figure 2). We wanted to focus on one sex to improve our sample sizes and in birds females are typically considered the choosier sex (Andersson, 1994; Andersson & Simmons, 2006), and in our four‐way trials there was no evidence of preference between red and yellow bills shown by males (Supporting Information; Table S3). In all two‐way trials, focal females were presented with one stimulus male with a yellow bill and one with a red bill. Focal females in ‘natural’ trials were presented with a choice between two males, one male of each subspecies with their natural bill colors. In order to control for other traits that might differ between subspecies (e.g., song), or to account for the differing reactions of a consubspecific male to a focal female, a separate set of trials were run in which focal females were presented with a choice between two males of their own subspecies (i.e., consubspecific males), however, both males had their bill color manipulated by nail polish, to be either artificial yellow or artificial red. We focused analyses on the proportion of time the focal bird spent in front of each stimulus bird, calculated as a proportion of the total time spent in front of any stimulus birds, because the absolute time could be skewed by certain trials with large absolute values. Nonetheless, because a longer absolute length of time spent may reflect increased preference, we also ran analyses using the absolute time and find similar results (Table A2).

We performed a total of 87 trials with stimulus birds having natural bill colors, 41 with an *acuticauda* female and 46 with a *hecki* female, each female given a single trial. Of these, 70 were successful (i.e., in 17 cases the focal female did not move for 15 min). Of the successful trials, 32 were with *acuticauda* females (64 different males were used, each used once) and 38 were with *hecki* females (70 different males used either once or twice; mean = 1.08, SD = 0.28). For the trials with artificial bill colors for the stimulus birds, fewer were performed due to the challenges associated with bill color manipulation. A total of 40 trials were performed using 16 *hecki* females and 24 *acuticauda* females. Of these, 36 were successful (i.e., in 4 cases the focal female did not move for 15 min). Of the successful trials, 13 had *acuticauda* females (11 different stimulus males were used) and 23 had *hecki* females (10 stimulus males were used).

### 2.5 Bill color inheritance in F_1_ hybrids

To evaluate evidence of bill color inheritance in hybrids, we measured the bill color of 110 captive bred first‐generation (F_1_) hybrids via reflectance spectrophotometry using the methods outlined above. F_1_ hybrids were produced by establishing mixed breeding pairs containing a male from a parental subspecies with a female from the other parental subspecies in small aviaries at Macquarie University. As one candidate locus for bill color is located on the Z chromosome (tetratricopeptide repeat protein coding gene *TTC39B*; Hooper et al., 2019; Lopez et al., 2021), we explicitly examined whether bill color differed based on the sex of the F_1_ hybrid and the subspecies identity of its parents.

### 2.6 Statistical methods

For statistical analyses we used R version 4.1.2 (R Core Team, 2020), using RStudio version 1.2.5033 (RStudio Team, 2020) for the graphical interface. We primarily used linear mixed models (LMMs) in the lme4 package (Bates et al., 2015), and where significant differences between groups were detected we assessed all pairwise combinations using the estimated marginal means (EMMs; emmeans package in R (Lenth, 2020)). We used the DHARMa package to examine residuals to check for normality and homogeneity of variance in our models (Hartig, 2021). For all models using proportion data, the proportion of time was logit transformed to stabilize variances, using the formula ln((p + 0.01)/(1‐p + 0.01)) where p is the proportion of time and 0.01 was added as there were zeros in the proportion data that cannot be transformed using the logit scale.

For the two‐way trials, we assessed the data in four groups: whether the focal female was *hecki* or *acuticauda*, and whether the bill color of stimulus males was natural or artificial. We performed linear mixed models to test whether the proportion of time spent with stimulus birds differed depending on stimulus bird bill color, with focal bird ID and trial number as random effects. For the trials with *acuticauda* focal females and natural bill colors, each stimulus and each focal bird was only used in one trial, so a linear model was applied instead (no random effects). In trials with yellow focal females and natural stimulus bill colors there are two extreme values, but excluding these points produces the same results, so we report results with the full dataset. As there were fewer manipulated trials, we calculated the power they had to detect any effect identified in the natural trials. We analyzed the four‐way trials using linear models in a similar fashion to the two‐way trials (details in the Supporting Information). To test for evidence of biased inheritance of bill color in F_1_ hybrids, we ran a linear model with the independent variable as H3 (hue) and sex and cross (and their interaction) as dependent variables.

## 3 RESULTS

**FIGURE 3:**
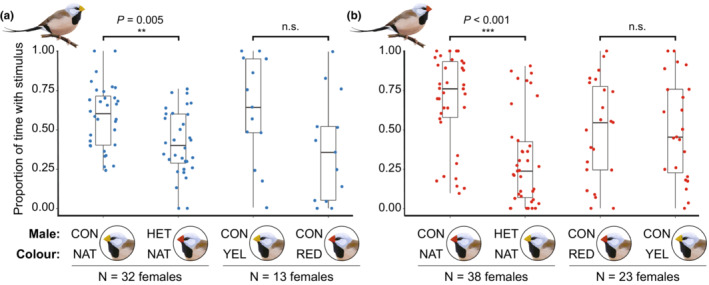
Female two‐way choice trials suggest an associative preference exists for males of their own subspecies. The proportion of time focal females of subspecies *acuticauda* (a) or *hecki* (b) spent with stimulus males of either subspecies with unmanipulated natural bill color (i.e., NATural CONspecifc or HETerospecifc choice) and stimulus males of their own subspecies with manipulated bill color (artificial yellow or red). The number of trials (i.e., focal females) is given beneath the results of each of our four experimental treatment groups.

**FIGURE A2:**
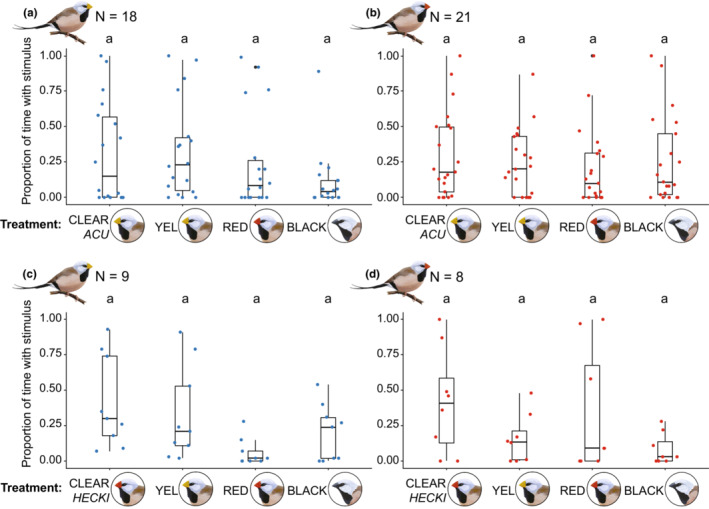
Male four‐way choice trials suggest no clear associative preference in long‐tailed finch males based upon female bill color. (A–D) The proportion of time focal males spent with each of the four different stimulus females is given for our four experimental setups. Panels (a) and (c) present trials conducted where the focal males were of subspecies *acuticauda* and panels (b) and (d) trials conducted where the focal males were of subspecies *hecki*. Panels (a) and (b) present results from trials in which males were presented with a choice between females with bills that were either natural *acuticauda* yellow (clear control), artificial yellow, artificial red, or artificial black. Panels (C) and (D) present results from trials in which males were presented with a choice between females with bills that were either natural *hecki* red (clear control), artificial yellow, artificial red, or artificial black. The number of trials for each experimental setup is shown and different letters indicate where statistical significance exists in treatment groups (*p* < 0.05; Tukey's HSD test).

**FIGURE A3:**
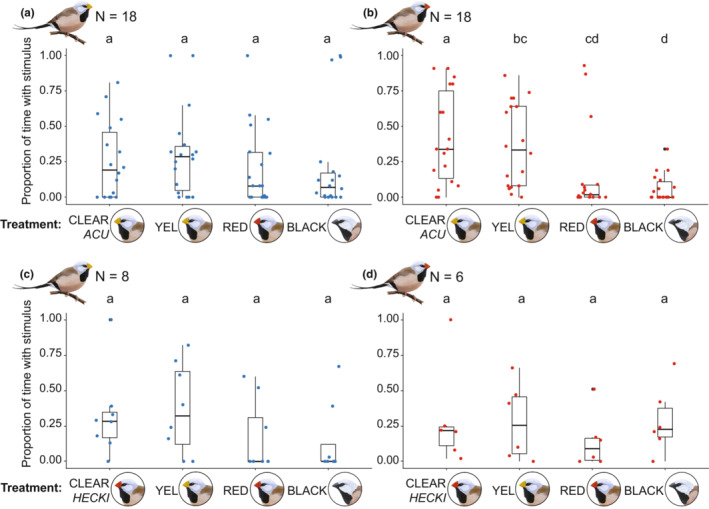
Female four‐way choice trials suggest potential associative preference in females of subspecies *hecki* for yellow bill color. (A–D) The proportion of time focal females spent with each of the four different stimulus males is given for our four experimental setups. Panels (a) and (c) present trials conducted where the focal females were of subspecies *acuticauda* and panels (b) and (d) trials conducted where the focal females were of subspecies *hecki*. Panels (a) and (b) present results from trials in which females were presented with a choice between males with bills that were either natural *acuticauda* yellow (clear control), artificial yellow, artificial red, or artificial black. Panels (C) and (D) present results from trials in which females were presented with a choice between males with bills that were either natural *hecki* red (clear control), artificial yellow, artificial red, or artificial black. The number of trials for each experimental setup is shown and different letters indicate where statistical significance exists in treatment groups (*p* < 0.05; Tukey's HSD test).

**FIGURE A4:**
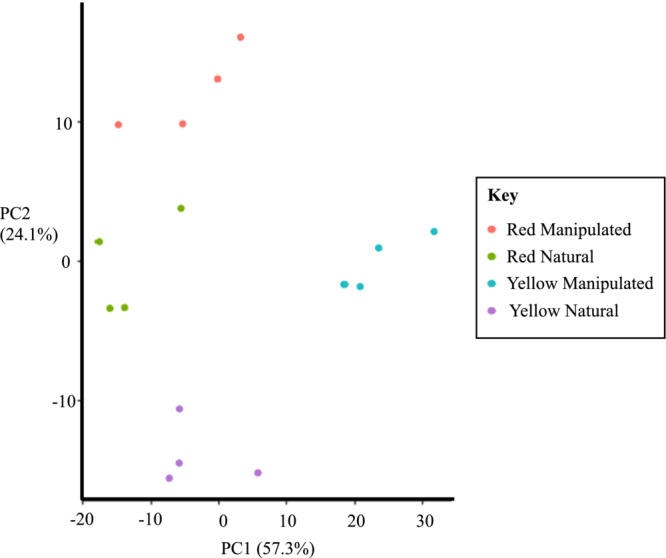
The first two principal components of spectrophotometry data for the natural and manipulated bill colors used in behavioral trials. Spectrophotometry data from the 300–700 nm range were smoothed and normalized before being input into a principal components analysis. The percentage of variance in the data explained by each components is in brackets.

In the two‐way trials in which the stimulus males had natural bill colors, females of subspecies *hecki* spent a significantly greater proportion of time with *hecki* than *acuticauda* males (2.2 times as long, χ^2^ = 24.47, Df = 74, *p* < 0.001; Figure 3b; Table S1) and females of subspecies *acuticauda* spent a significantly greater proportion of time with *acuticauda* males than *hecki* males (1.4 times as long, χ^2^ = 7.93, Df = 62, *p* = 0.005; if we exclude 2 trials where females spent entire time with one male that appear as outliers after logit transformation, we get qualitatively the same results χ^2^ = 6.37, Df = 58, *p* = 0.012; Figure 3a). In two‐way trials with artificial bill colors, for *acuticauda* females the same pattern held but was borderline nonsignificant (χ^2^ = 2.89, Df = 11, *p* = 0.089; Figure 3a), and there was no significant difference for *hecki* females (χ^2^ = 0.281, Df = 44, *p* = 0.59; Figure 3b; Table S1). The borderline nonsignificant result in the manipulated bill color trials by *acuticauda* females (*p* = 0.089) may be driven by the much smaller sample size than natural trials (*N* = 13 vs. 32 trials) resulting in low statistical power to identify a similar sized effect (effect size of natural trials d = 0.67, power of artificial trials = 0.38). In the four‐way trials, we observed no clear evidence for mate preference by males or by *acuticauda* females, and although *hecki* females showed a preference for yellow bills, this was only in trials with a clear yellow bill (and without a clear red bill; Figures A2, A3; Table A3).

**FIGURE 4:**
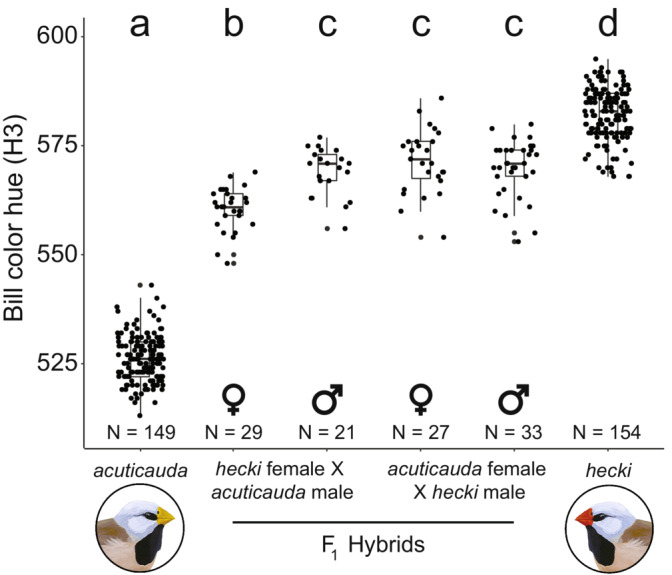
Bill color of first‐generation (F_1_) hybrids is dependent on hybrid sex and the direction of hybrid cross. Variation in bill color hue (H3) is shown for long‐tailed finches from subspecies *acuticauda* and *hecki* (results from both sexes) and their F_1_ hybrids grouped by the direction of hybrid cross (*hecki* female x *acuticauda* male or *acuticauda* female × *hecki* male) and sex (female or male). All F_1_ hybrids have intermediately ‘orange’ colored bills, however, F_1_ hybrid females with *acuticauda* fathers have significantly yellower bills than F_1_ hybrid females with *hecki* fathers or either class of F_1_ hybrid male. Different letters indicate statistical significance between treatment groups (*p* < 0.05; Tukey's HSD test).

When examining the dominance of genes underlying bill color, we found that the bill color hue (H3) of F_1_ hybrids was significantly affected by the interaction between sex and cross direction (*F* = 18.17, Df = 3, *p* < 0.001): F_1_ females with an *acuticauda* father and a *hecki* mother had significantly yellower bills than any of the other groups (*p* < 0.005 for all; Figure 4; Table A1). There was no difference between males of either cross direction and females with a *hecki* father and an *acuticauda* mother (*p* > 0.99 for all; Figure 4; Table A1).

## 4 DISCUSSION

In this study, we used behavioral assays to evaluate mate choice based on bill coloration in both subspecies of the long‐tailed finch (*Poephila acuticauda*). We found no evidence for a common female preference for yellow billed individuals across both subspecies that could drive the observed introgression of yellow bill color in the wild. Our results instead suggest that females have a weak assortative preference for their own subspecies, whereby *acuticauda* females prefer naturally yellow‐billed males and *hecki* females prefer naturally red‐billed males. However, this assortative preference weakens or disappears when females are exposed to two males of their own subspecies with bills manipulated to be red or yellow, suggesting that the assortative preference is multimodal or based on another trait that differs between the subspecies (e.g., song). A potential source of the assortative mate preference observed in this study is sexual imprinting, whereby offspring develop their mate preferences by learning during a short sensitive period, based on the phenotypes of the parents that reared them (Brodin & Haas, 2006; Clayton, 1993; Irwin & Price, 1999; Lorenz, 1937). Sexual imprinting has widespread documentation in birds and has received considerable support as a driver of mate preferences in the Estrildid finches (Cate, ten, & Vos, 1999; Clayton, 1990; Oetting & Bischof, 1996; Witte & Sawka, 2003).

Intriguingly we found that all F_1_ birds had bill color hue closer to red *hecki* bills than yellow *acuticauda* bills, but that F_1_ females with an *acuticauda* father had significantly yellower bills than other F_1_ hybrids. This is consistent with one candidate gene for bill color being located on the Z chromosome (female birds have only one copy of the Z chromosome, that is, are hemizygous) as found in Hooper et al. (2019). As female hybrids with *hecki* fathers did not have significantly redder bills than any other hybrid group (Figure 4), it suggests that the *acuticauda* contribution to bill color is at least partially recessive to the *hecki* contribution on the Z chromosome. The hybrid zone of the European crow provides the best understood case of assortative mating‐driven introgression, for which simulation models suggest that upon secondary contact the dominance of dark plumage alleles over light ones resulted in high frequency of dark plumage among hybrids (Metzler et al., 2021). The models predict that assortative mating preference then induced positive frequency‐dependent selection on dark morphs, increasing the alleles underpinning dark plumage in the hybrid zone, moving the dark plumage into the light plumage lineage (Knief et al. 2019; Metzler et al., 2021). Another study on assortative‐mate preference‐driven introgression also performed forward simulations that suggested that the direction of introgression can be variable and stochastic, such that in 33% of their simulations it was the recessive phenotype that introgressed into the dominant phenotype lineage (Semenov et al., 2021). This appears to be the case with the recessive dark head plumage of *Motacilla alba personata* into *M. a. alba* (Semenov et al., 2017, 2021)*,* and in the present study with the introgression of the recessive yellow bill color. The simulations by Semenov et al. (2021) also suggest that relatively weak mate preference, as observed in the current study (Figure 3), can be enough to contribute to the unidirectional introgression of phenotypic traits (Metzler et al., 2021; Semenov et al., 2021). Developing a better understanding of the genetic architecture underlying bill color is now a priority in the long‐tailed finch.

Our dataset suggests that there is assortative mate preference for bill color when focal birds are presented with natural bill‐colored males, but less so, or not at all, for trials with manipulated bill color. *P. acuticauda* females showed the same pattern of assortative preference for manipulated bills, but the result was borderline nonsignificant, potentially due to the much lower statistical power resulting from the small sample size (Figure 3). *P. hecki* females showed no pattern of assortative preference for manipulated bills (Figure 3), which may be because the artificial red bill colors were not convincing replicates of the natural red bills, as reflected by the H3 values (Figure 1). As such it remains possible that the assortative preference observed in trials with natural bill colors is truly based on bill color itself, but that we did not find convincing evidence of this due to either low statistical power and/or the slightly unnatural bill color manipulation (e.g. in the UV component). Alternatively, this discrepancy might instead suggest that the assortative preference observed in these data may actually be due to a combination of traits that differs between subspecies such as song (Knerr et al. in prep; Zann, 1976), chemical signals (Van Huynh and Rice, 2019; 2021), or other aspects of courtship behavior or parental care investment. Alternatively, as shown in the closely related zebra finch *Taeniopygia guttata*, the assay of female choice using this type of approach might be influenced by the behavior of the stimulus bird, which may depend on the subspecies of both male and female involved (Clayton, 1990; Rutstein et al., 2007). This is difficult to address and would require also manipulating the appearance of the focal female.

In conclusion, we find evidence that females of both subspecies of long‐tailed finch appear to have an assortative mate preference for males of their own subspecies but did not find evidence for assortative preference based on artificially manipulated bill color alone. We also found that bill color of F_1_ hybrid females was significantly influenced by the subspecies identity of their father. This raises the possibility that assortative mate preference, in combination with dominant effects of underlying genes, may be driving the introgression of bill color in this system, in line with emerging evidence in other avian systems. While unidirectional introgression of sexual character traits is a common phenomenon in wild hybrid zones, often leading to speculation as to the proximate causes, our results highlight the need to experimentally validate these assumptions. We hope to further motivate greater efforts to understand the genetic basis of rapidly diverging coloration and other signaling traits.

## AUTHOR CONTRIBUTIONS

**Callum S. McDiarmid:** Conceptualization (Lead); Data curation (Lead); Formal analysis (Lead); Funding acquisition (Supporting); Investigation (Supporting); Visualization (Supporting); Writing – Original Draft (Lead); Writing – Review and Editing (Equal). **Fiona Finch:** Investigation (equal); methodology (equal); writing – review and editing (supporting). **Marianne Peso:** Investigation (equal); methodology (equal); writing – review and editing (supporting). **Erica van Rooij:** Investigation (equal); methodology (equal); writing – review and editing (supporting). **Daniel Marc Hooper:** Conceptualization (equal); formal analysis (supporting); visualization (lead); writing – original draft (equal); writing – review and editing (equal). **Melissah Rowe:** Conceptualization (equal); formal analysis (supporting); writing – original draft (equal); writing – review and editing (equal). **Simon Griffith:** Conceptualization (equal); funding acquisition (lead); investigation (equal); methodology (equal); project administration (equal); writing – original draft (equal); writing – review and editing (equal).

## CONFLICT OF INTEREST STATEMENT

The authors declare no conflicts of interest.

## Supporting information


Supporting information S1.

Supporting information S2.

## Data Availability

Data files and the corresponding R script have been uploaded to the Open Science Framework and are publicly available (https://osf.io/9xwqd/; DOI: 10.17605/OSF.IO/9XWQD).

## APPENDIX A

**TABLE A1:** Pairwise contrast among F1 groups (sex and parental cross), given the significant effect of sex*cross on bill color (H3).

Sex_1	Mum_1	Dad_1	Vs	Sex_2	Mum_2	Dad_2	Estimate	SE	Df	t.ratio	*p*.Value
F	hec	acu	‐	M	hec	acu	**−8.544**	**1.75**	**106**	**−4.874**	**<0.0001**
F	hec	acu	‐	F	acu	hec	**−10.93**	**1.64**	**106**	**6.681**	**<0.0001**
F	hec	acu	‐	M	acu	hec	**−9.145**	**1.56**	**106**	**5.873**	**<0.0001**
F	acu	hec	‐	M	acu	hec	1.785	1.59	106	1.124	0.6755
F	acu	hec	‐	M	hec	acu	2.386	1.78	106	1.341	0.5393
M	acu	hec	‐	M	hec	acu	0.602	1.71	106	0.352	0.9849

**TABLE A2:** Model outputs for two‐way female choice trials, comparing the absolute length of time focal females spent with each of the red and yellow stimulus male birds.

Focal col	Artificial	N trials	χ^2^	Df	*p*‐Value
Red	Y	23	0.23	44	0.63
Red	N	38	**12.49**	**66**	<0.001
Yel	Y	13	3.79	24	0.052
Yel	N	32	**6.87**	**62**	0.009

*Note*: Focal Col = the bill color (and subspecies) of the choosing bird, Artificial = whether bill colors of the stimulus birds all had their bill colors manipulated (Y) or none of them did (N).

**TABLE A3:** Four‐way mate‐choice trials considering the proportion of time spent with each stimulus bird, with Trial ID as a random effect.

							Average proportion of time spent (SD). Different letters (i.e., A and B) indicate significant difference among groups.					
Focal sex	Focal col	Clear	χ^2^	Df	*p*‐Value	N trials	Black	Clear red	Clear yellow	Red	Yellow	
Male	Red	ClearR	3.12	28	0.37	8	0.08 (0.11)	0.42 (0.37)		0.34 (0.44)	0.16 (0.17)	
	Red	ClearY	1.01	80	0.8	21	0.26 (0.31)		0.30 (0.30)	0.20 (0.27)	0.24 (0.24)	FocalID included
	Yell	ClearY	5.32	68	0.16	18	0.11 (0.21)		0.31 (0.36)	0.25 (0.35)	0.33 (0.34)	FocalID included
	Yell^a^	ClearR	**14.868**	**32**	**0.002**	**9**	0.20 (0.20) A	0.41 (0.32) A		0.06 (0.09) B	0.33 (0.33) A	
Female	Red	ClearR	2.78	20	0.44	6	0.29 (0.24)	0.29 (0.36)		0.14 (0.19)	0.28 (0.27)	
	Red	ClearY	**27.16**	**68**	**<0.001**	**18**	0.06 (0.10) A		0.42 (0.33) B	0.15 (0.30) A	0.37 (0.30) B	FocalID included
	Yell	ClearY	1.92	68	0.59	18	0.22 (0.36)		0.26 (0.27)	0.22 (0.28)	0.31 (0.31)	FocalID included
	Yell^b^	ClearR	**8.05**	**28**	**0.045**	**8**	0.14 (0.26)	0.32 (0.30)		0.17 (0.26)	0.37 (0.32)	

*Note*: Due to the low sample sizes some additional potentially confounding factors could not be statistically accounted for, including the genetic lineage of stimulus birds, and that stimulus birds were included in variable numbers of trials.

^a^ Note that here there is a significant difference, but driven by a non‐preference for particular group/s, and no obvious preference for a particular phenotype.

^b^ Note that here the *p*‐value was <0.05 for the main model, but the post hoc tests of all pairwise comparisons found no significant differences between groups.

**TABLE A4:** Results from principal component analysis (PCA) that was run on spectrophotometry data between 300 and 700 nm, including red (R) and yellow (Y), manipulated (Manip) and natural (Nat) bill colors.

ID	PC1	PC2	PC3	PC4	PC5	PC6	PC7	PC8	PC9	PC10	PC11	PC12	PC13	PC14	PC15	PC16	PC17
73787.RNat	−17.673725	1.42302908	0.98380176	5.69086952	0.37947994	0.92276669	1.64171805	−1.104018	−0.1291188	0.02871076	0.18851486	−0.0530893	−0.0479923	0.0403673	−0.0335556	−0.024829	8.73 E‐16
73792.RNat	−13.882909	−3.312158	9.8437255	−2.5616206	1.15077949	1.59110701	−0.3333626	0.50522464	−0.3621537	0.18630401	−0.0117937	−0.0777458	0.01629919	0.06489799	0.03017557	0.03255748	−4.57 E‐15
73793.RNat	−5.5334991	3.7856516	−1.5673562	−1.6122213	−2.7460325	4.51797183	2.24656837	−0.0709298	−0.0091277	−0.1653588	−0.1330695	0.05809541	0.0190761	−0.031084	0.02777083	−0.0018075	−5.53 E‐15
73794.RNat	−16.082	−3.384035	12.3630856	−1.4937559	4.33149389	1.06235812	−0.9313055	0.05066043	0.25350477	0.00571899	0.0104596	0.01791098	−0.0223835	−0.0788042	−0.0275504	−0.0018087	−4.56 E‐15
73912.RManip	−5.3420395	9.83614046	−4.8548226	−0.7691149	−2.4674896	−0.0466632	−0.4666527	1.15554607	−0.5187555	0.19093154	−0.1142782	−0.0142325	0.07284502	−0.0097989	−0.059164	−0.0093834	−3.33 E‐15
73915.RManip	3.28849948	16.0974803	−6.1320321	−4.2246485	−0.243939	1.70592072	−1.5129456	0.74388902	0.69546171	−0.0647994	0.13719055	−0.0476127	−0.0661102	0.0287186	0.00302563	−0.010443	−4.95 E‐15
73946.YManip	18.5229432	−1.6721654	5.03237419	0.94198768	−2.6255771	−1.3628539	1.22437963	0.7823282	−0.0142778	0.11530235	0.05467287	0.11969088	−0.1455209	−0.0047267	−0.0141858	0.02419867	−1.40 E‐15
73950.YManip	31.7671852	2.14690701	0.77269593	2.20124898	1.70140844	2.10929882	−1.8269403	−1.3264723	−0.2688024	0.13330779	−0.1296356	0.07725482	−0.0119672	0.01615201	0.0028963	−0.0127849	−1.18 E‐15
73951.YManip	20.8068593	−1.8331764	4.33806422	1.63629598	−2.0750272	−0.8257798	0.11041936	0.33770554	−0.2333241	0.06449946	0.14864832	−0.177788	0.02475984	−0.0514968	0.03826421	−0.0210748	1.81 E‐15
73953.YManip	23.5898281	0.97434297	5.79416882	2.39151629	2.15050264	−0.752936	1.2307294	0.37593526	0.47731325	−0.2490056	−0.0424277	−0.0302178	0.11685041	0.03163889	−0.0274805	0.01308991	7.64 E‐15
73972.RManip	−14.810393	9.77063443	−4.5527343	7.37786556	0.44797298	−2.0799951	−0.0608997	−0.1992219	0.4300318	0.13319075	−0.2468178	−0.0609906	−0.0322177	−0.0147239	0.02616788	0.01346273	5.43 E‐15
73973.RManip	−0.1658124	13.073736	−0.6582157	−9.2624253	1.27194234	−3.3126493	1.18098906	−1.3776894	−0.161441	0.03150181	0.00418691	0.00891627	0.01308565	−0.0058527	0.00589252	0.00266745	−5.73 E‐15
G111.YNat	5.7008383	−15.185654	−17.554858	−0.7954141	4.98410603	0.51465272	0.64702337	0.40843101	−0.2386411	−0.0062302	0.04150285	−0.0395287	−0.0261429	−0.014717	−0.0017093	0.01474375	−5.76 E‐15
G124.YNat	−5.833474	−14.498435	−4.0927215	−0.5113627	−3.4869112	−0.0454157	−0.531598	−0.714537	0.59206623	0.26357276	0.13952708	0.07887384	0.10144362	−0.0048903	0.00045242	0.01054477	−1.63 E‐15
G156.YNat	−5.7189411	−10.625308	−0.5031829	−1.0091836	−4.0744203	−0.736532	−1.6671774	−0.8024464	−0.193889	−0.3899622	−0.075303	−0.0825227	−0.0461271	0.00121504	−0.019651	0.01727194	−2.20 E‐15
G168.YNat	−7.2720917	−15.55662	2.0695584	−2.3832807	0.33645788	−1.9595588	0.12644721	0.77677877	0.11787543	−0.0412849	−0.1543658	0.06318689	−0.0186707	0.03601379	0.01824295	−0.0501901	−1.82 E‐15
