# Supplementary material for: Experimentally testing mate preference in an avian system with unidirectional bill color introgression
Source: Ecol Evol. 2023 Feb 21;13(2):e9812. doi: 10.1002/ece3.9812 (PMC9942114; doi:10.1002/ece3.9812)
Supplement: Supplementary file 1 — Supporting information S1. [file ECE3-13-e9812-s001.docx]

Supplementary Materials

Methods

Four-way trials

As outlined in the main text, we focus our analysis and enterpretation on the set of two-way trials performed, but here also report the methods and results for the four-way trials for completeness and transparency. There were a total of 60 four-way mate choice trials performed with 30 focal males (two trials per male), 32 trials with *hecki* males and 28 with *acuticauda* males. Eight trials were not assessed due to non-responsive focal birds. Of the 56 successful trials, the focal male was of subspecies *acuticauda* in 27 trials (23 males) and of subspecies *hecki* in 29 trials (22 males). A total of 40 different female stimulus birds were used and each individual was used on average in 5.6 trials (SD = 0.5, range = 5-6), and for each trial all stimulus birds were genetically of the same subspecies (with a few exceptions when one *acuticauda* female was included with an otherwise *hecki* group). The subspecies breakdowns of these 40 stimulus females that had their bills painted was: 10 painted yellow (8 *acuticauda*, 2 *hecki*), 10 red (7 *acuticauda*, 3 *hecki*), 10 black (*7 acuticauda,* 3 *hecki*), and 10 clear (7 *acuticauda*, 3 *hecki*).

Similarly, 60 four-way mate choice trials with 30 focal females were performed (two trials per female), of which 10 were aborted due to non-responsive focal birds. Of the 50 successful trials, the focal female was from subspecies *acuticauda* in 26 trials and from subspecies *hecki* in 24 trials. We used a total of 40 different male stimulus birds and each individual was used in an average of five trials (SD = 1.6, range = 2-8). The subspecies breakdowns of these 40 stimulus males that had their bills painted was: 10 painted yellow (6 *acuticauda,* 4 *hecki*), 10 red (6 *acuticauda,* 4 *hecki*), 10 black (6 *acuticauda,*4 *hecki*), and 10 clear (8 *acuticauda*, 2 *hecki*).

For our analyses, we first plotted our data to explore patterns, and it appeared that different trial contexts may influence animals preference, we analysed each group separately. For the 4-way trials this meant four groups; either *hecki* or *acuticauda* focal bird and either *hecki* or *acuticauda* ‘clear’ stimulus bird. For each group we fitted a linear mixed models to test whether the proportion of time spent with stimulus birds differed depending on stimulus bird bill colour, with focal bird ID and Trial Number as random effects.

Results

**Male Choice 4-way**

Full model outputs can be seen in Tables S3 and S4. Focal males in the 4-way trials only showed a preference when the focal males were *acuticauda* and the clear artificial bill was red (χ^2^ = 15.1, DF = 3, *p* = 0.0017; Figure S2), spending less time with the artificial red bill than either the clear red bills (t-ratio = -3.79, DF = 24, *p* = 0.005) or artificial yellow bills (t-ratio = 3.08, DF = 24, *p* = 0.025).

**Female choice 4-way**

Focal females in the 4-way trials only showed a significant preference when *hecki* focal females were exposed to clear yellow bills (χ^2^ = 27.24, DF = 3, *p* < 0.001; Figure S3), where they spent more time clear yellow bills than either black (t-ratio = 4.3, DF = 51, *p* <0.001) or artificial red (t-ratio = 3.8, DF = 51, *p* = 0.0023) and spent more time with artificial yellow than either black (t-ratio = 4.1, DF = 51, *p* < 0.001) or artificial red (t-ratio = 3.6, DF = 51, *p* = 0.004).
